# Jordan's 2002 to 2012 Fertility Stall and Parallel USAID Investments in Family Planning: Lessons From an Assessment to Guide Future Programming

**DOI:** 10.9745/GHSP-D-17-00191

**Published:** 2017-12-28

**Authors:** Esther Spindler, Nisreen Bitar, Julie Solo, Elizabeth Menstell, Dominick Shattuck

**Affiliations:** a Institute for Reproductive Health, Georgetown University, Washington, DC, USA.; b Independent consultant, Amman, Jordan.; c Independent consultant, Durham, NC, USA.

## Abstract

Health practitioners, researchers, and donors are stumped about Jordan's stalled fertility rate, which has stagnated between 3.7 and 3.5 children per woman from 2002 to 2012, above the national replacement level of 2.1. This stall paralleled United States Agency for International Development (USAID) funding investments in family planning in Jordan, triggering an assessment of USAID family planning programming in Jordan. This article describes the methods, results, and implications of the programmatic assessment. Methods included an extensive desk review of USAID programs in Jordan and 69 interviews with reproductive health stakeholders. We explored reasons for fertility stagnation in Jordan's total fertility rate (TFR) and assessed the effects of USAID programming on family planning outcomes over the same time period. The assessment results suggest that the increased use of less effective methods, in particular withdrawal and condoms, are contributing to Jordan's TFR stall. Jordan's limited method mix, combined with strong sociocultural determinants around reproduction and fertility desires, have contributed to low contraceptive effectiveness in Jordan. Over the same time period, USAID contributions toward increasing family planning access and use, largely focused on service delivery programs, were extensive. Examples of effective initiatives, among others, include task shifting of IUD insertion services to midwives due to a shortage of female physicians. However, key challenges to improved use of family planning services include limited government investments in family planning programs, influential service provider behaviors and biases that limit informed counseling and choice, pervasive strong social norms of family size and fertility, and limited availability of different contraceptive methods. In contexts where sociocultural norms and a limited method mix are the dominant barriers toward improved family planning use, increased national government investments toward synchronized service delivery and social and behavior change activities may be needed to catalyze national-level improvements in family planning outcomes.

## Abstract (precis)

Jordan's limited method mix, which has shifted toward less effective methods such as withdrawal and condoms, is a likely contributor to the plateau, coupled with social and cultural norms that discourage contraceptive use, such as preference for large family size and pressure to have a child immediately after marriage. Greater investment in social and behavior change and advocacy for stronger programming efforts are warranted.

## BACKGROUND

The total fertility rate (TFR) in Jordan plateaued between 2002 and 2012, despite nearly universal knowledge of contraceptive methods, high rates of female literacy, support for spacing births, and several years of increases in the contraceptive prevalence rate (CPR). After a steep drop in TFR from 6.6 to 3.7 children per woman between 1983 and 2002, the following decade saw only a slight decline from 3.7 to 3.5.^1^ Such a period of little to no decline in the fertility rate in countries in transition to replacement-level fertility (2.1 children per woman) is usually referred to as a “stall” in fertility. A 2012 analysis of Jordanian fertility patterns found that the stall was not due to data errors, and it was one of the longest-lasting periods of stagnation assessed worldwide.^2^

The fertility stall in Jordan between 2002 and 2012 was one of the longest-lasting periods of stagnation assessed worldwide.

A TFR stall is typically accompanied by stalls in multiple parallel measures, including the modern contraceptive prevalence rate (mCPR), unmet need and demand for family planning, and the desired fertility rate, all of which have also stalled in Jordan.^3^ From 2002 to 2012, Jordan's CPR increased from 56% to 61%. However, the increase was primarily due to use of traditional methods, which rose from 15% to 19%. The mCPR increased only marginally over the same period (41% to 42%).^1^^,^^4^ Unmet need for family planning is relatively low in Jordan, hovering between 11% and 12% from 2002 to 2012. However, if individuals using traditional methods are classified as having an unmet need, the met demand for family planning drops from over 80% to about 60%, below the global target of 75%.^1^

Jordan's stalled TFR of 3.5 children per woman poses challenges for the country's population and development goals. Jordan is a small country, about the size of the U.S. state of Maine, with scarce water and environmental resources. At the same time, Jordan is experiencing a population boom, from 5.3 million people in 2004 to 9.5 million people in 2015.^5^^,^^6^ The population growth is in part due to a migration influx. In 2015, about one-third of Jordan's population was non-Jordanian, with approximately 1.3 million Syrians, 636,000 Egyptians, 634,000 Palestinians, and 130,000 Iraqis, among other nationalities.^7^^,^^8^ To ease the pressures of massive population growth and scarce natural resources, Jordan's national family planning goal is to reach a replacement level of fertility by 2030.^7^

Jordan receives a sizable assistance package from the U.S. government for economic and development programs. From 2002 to 2012, we estimated that the United States Agency for International Development (USAID) obligated close to US$307 million in Jordan's health sector, with the majority focused on maternal and child health, and reproductive health programming.^8^ This USAID investment paralleled the fertility stagnation, prompting the USAID/Jordan Mission to request an external assessment of their family planning programs. This article describes key insights from the assessment.

During the fertility stall, USAID obligated close to US$307 million in Jordan's health sector.

## METHODS

A programmatic assessment of USAID-funded reproductive health programs in Jordan was conducted between October 2015 and March 2016. Our evaluation team reviewed program documentation that included primary USAID project reports, briefs, external evaluations, and studies, and conducted interviews with key stakeholders. The assessment focused on 3 research questions:

1. What factors contributed to the TFR and mCPR stagnation in Jordan?
2. What projects did USAID funding support during the stagnation and what impact was attributed to those projects?
3. What insights were gained from earlier programming that can guide USAID's future reproductive health strategy in Jordan?^8^

To answer these research questions, assessment methods included a desk review of more than 83 documents and 69 interviews with 168 participants—including 23 U.S.-based key informants and 145 respondents in Jordan.

### Desk Review

We reviewed multiple data sources, including 30 project reports, 42 external studies and evaluations, and 11 project briefs. The documents were identified through the USAID Development Experience Clearinghouse and online journal searches, and provided by USAID/Jordan staff. Two evaluation team members reviewed the documentation and entered the data in a desk review matrix, categorized by type of documentation, project, funding amount, geographic coverage, time period, target population, type of activities, evaluation methods, key outcomes, and recommendations. We identified a total of 20 USAID-funded projects focused on reproductive health and family planning outcomes over a 20-year period (1995 to 2015). To understand the USAID portfolio distribution over time, we categorized the projects according to 3 different funding streams: service delivery, policy and advocacy, and social and behavior change (SBC) programming.

### Key Informant Interviews

We developed a semistructured questionnaire guide for key informant interviews that generated information about each of the 3 research questions. Data were analyzed by comparing salient themes across the following groups of key informants: (1) central Ministry of Health (MOH) officials, (2) regional MOH officials, (3) private-sector health professionals, (4) other government officials, (5) donor staff, (6) international experts, and (7) USAID project contractors (Table 1). We entered interview transcripts and notes in Dedoose, a qualitative data analysis software, and systematically coded them in accordance with the research questions. Coded excerpts were subsequently exported to Microsoft Excel for thematic analysis. The team conducted qualitative salience analysis by following these 2 steps: (1) aggregating the frequency that a thematic code was applied within a given transcript (e.g., number of times “son preference” is coded as a factor of TFR stagnation), and (2) dividing the total aggregate number of times a thematic code was applied by the number of transcripts for that given key informant group. The resulting salience score was the mean frequency of each theme calculated for that given key informant subgroup. A higher salience score represented greater relative importance of the theme within that subgroup of key informants.^9^

**TABLE 1.** Key Informant Interview Groups (N=69)

Interview Group	No. of Interviews
**Central MOH officials**	11
*MOH administrative employees in Amman*	
**Regional MOH officials**	17
*MOH employees in regional hospitals and health centers*	
**Private-sector health professionals**	6
*Physicians, outreach workers, and NGO project directors in the private sector*	
**Other government officials** *Officers at non-MOH government departments (e.g., Higher Population Council, Royal Medical Services)*	10
**Donor staff**	6
*Staff at donor organizations (e.g., USAID, UNFPA, JICA, WHO)*	
**International experts**	9
*Experts in family planning familiar with, but working outside of, Jordan*	
**USAID project contractors**	10
*Contractors implementing USAID family planning projects in Jordan*	

Abbreviations: JICA, Japan International Cooperation Agency; MOH, Ministry of Health; UNFPA, United Nations Population Fund; USAID, United States Agency for International Development; WHO, World Health Organization.

One limitation of this analysis is the lack of a current national assessment of TFR and mCPR trends in Jordan. The most recent national-level data available are from the 2012 Jordan Demographic and Health Survey (DHS). To address this gap in the data, we collected couple-years of protection (CYP) data from the Jordanian MOH online database from 2010 to 2015. In addition, our findings are in part derived from interview data that reflect the views and belief systems of those stakeholders. We recognize that information shared by key informants may reflect biases related to the positive outcomes of their projects and experiences. The salience analysis, described above, aimed to reduce these potential biases by systematically analyzing and scoring interview data. Similarly, USAID's end-of-project reports tend to be biased toward positive outputs related to key milestones and successes, whereas less successful programmatic activities receive less representation in the reports. The evaluation team addressed these biases by triangulating data from multiple sources, including study briefs and external evaluations whenever available.

Finally, conducting an assessment of 20 years' worth of programming is a feasible but large undertaking. Given the diversity of intervention approaches and evaluation methodologies, we cannot attribute a quantitative change, or lack thereof, in national-level TFR and mCPR outcomes to USAID programmatic efforts. Rather, we situate the USAID programming efforts in the context of the fertility stall, providing an assessment methodology and programmatic case study for other contexts facing similar population and family planning issues.

## FINDINGS

### What Is Causing the TFR Stall in Jordan?

Documentation on Jordan's TFR stall exists, and evidence suggests that the increased use of less effective methods is a key contributing factor.^2^^,^^10^ Al-Massarweh^10^ applied Bongaarts' classic framework on the proximate determinants of fertility to Jordan,^11^ and found that the increase in CPR was not enough to offset the increase in proportion of married women during the same time period, as most of the increase was attributed to use of less effective traditional methods.^10^ Rashad and Zaky^12^ found similar TFR stalling trends over the same time period in Egypt, Jordan, and Syria. They suggested that in Jordan, use of contraception—and traditional methods in particular—played a stronger role than other proximate determinants (e.g., age at first marriage). The authors conclude that addressing women's concerns about side effects and improving the method mix could potentially shift women's use of traditional methods toward more effective modern methods.

Increased use of traditional contraceptive methods in Jordan played a strong role in the fertility stall.

Sociocultural and environmental factors, such as fear of side effects and limited method mix, are influencing the types of methods used in Jordan. The average salience scores from key informant interviews showed that key informants regarded these sociocultural factors—typically referred to as “indirect determinants” of fertility under Bongaarts' framework^11^—as key contributor factors of the stalled fertility in Jordan. As shown in Table 2, commonly cited factors among the interviewees for TFR stagnation included the desire for large families, fear of side effects with modern method use, influence of husbands and mothers-in-law, and preference for sons. Key informant interviews also cited issues related to Jordan's limited method mix and method stock-outs, in addition to provider behavior and bias, albeit less frequently. Overall, the assessment findings suggest that Jordan's limited method mix, coupled with social and cultural determinants of contraceptive use, may be influential factors of Jordan's stalled fertility outcomes, both of which are further explored in the sections that follow.

**TABLE 2.** Average Salience Scores for Reasons for TFR Stagnation in Jordan, by Interview Group

	Desire for Large Families	Fear of Side Effects With Modern Methods	Influence of Husbands and Mothers-in-Law	Preference for Sons	Short Birth Intervals; Rapid Pregnancy After Marriage
Central MOH officials (n=11)	2	1	0	0	0
Regional MOH officials (n=17)	7	12	6	4	2
Private sector health professionals (n=6)	4	3	2	3	1
Other government officials (n=10)	7	5	2	2	3
Donor staff (n=6)	3	1	3	1	0
International experts (n=9)	5	1	1	3	1
USAID project contractors (n=10)	3	6	3	1	2
**Average salience score, all key informants** (N=69)	**4.4**	**4.1**	**2.4**	**2.0**	**1.3**

Abbreviations: MOH, Ministry of Health; TFR, total fertility rate; USAID, United States Agency for International Development.

Factors such as the desire for large families, fear of side effects, influence of family, and preference for sons also influence use of contraception.

#### Limited Method Mix

**FIGURE 1.**
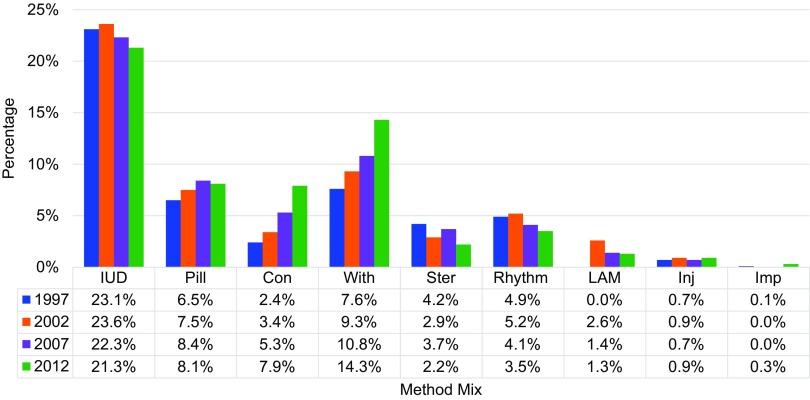
National Contraceptive Method Mix Among Contraceptive Users in Jordan by Year, 1997–2012 Abbreviations: Con, condoms; Imp, contraceptive implant; Inj, injectable contraception; IUD, intrauterine device; LAM, lactational amenorrhea method; Pill, oral contraceptive pills; Rhythm, rhythm or calendar method; Ster, female or male sterilization; With, withdrawal. Data from Jordan's Demographic and Health Surveys.

Jordan provides a context where family planning access and use are relatively high, but the diversity of methods available to clients is low. In recent years, the method mix has shifted toward less effective methods such as withdrawal and condoms. As shown in Figure 1, withdrawal use increased from 9.3% to 14.3% between 2002 and 2012, whereas more effective methods such as the intrauterine device (IUD) decreased from 23.6% to 21.3%.

**FIGURE 2.**
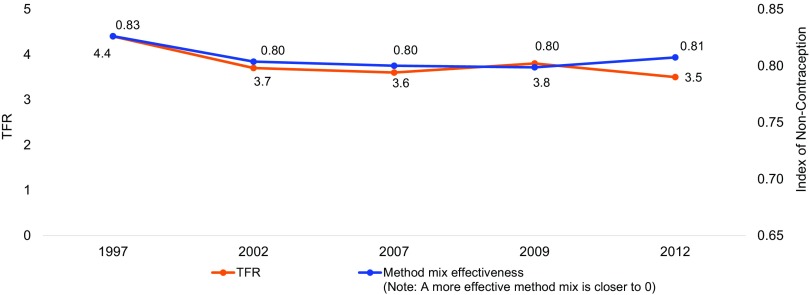
Changes in TFR and Contraceptive Method Mix Effectiveness^a^ in Jordan, 1997–2012 Abbreviation: TFR, total fertility rate. ^a^ Based on Bongaarts' index of non-contraception,^11^^,^^13^ which gauges the effectiveness of the method mix in relation to the prevalence of each method in a particular country. Index values range from 0 to 1.0, with values closer to 1.0 indicating a *less* effective method mix. Data from Jordan's Demographic and Health Surveys.

To better understand the impact of Jordan's limited method mix and shift in method use, we applied John Bongaarts' “index of non-contraception” to quantify the changing contraceptive method mix in Jordan.^11^^,^^13^ This index provides a way to gauge the effectiveness of the method mix in relation to the prevalence of each method in the target country. Index figures closer to 1.0 reflect a *less* effective method mix. To put this in perspective, Bongaarts provides the example of the United States' score improvement from 0.31 in 1965 to 0.22 in 1973, which represents substantial increases in availability of different family planning methods in less than 10 years. Figure 2 shows that over the 15 years from 1997 to 2012, the index score in Jordan decreased only two-tenths of a point (from 0.83 to 0.81), suggesting a method mix with limited effect.

This limited method mix, combined with women and men's concerns about side effects, is a likely contributor to Jordan's high rates of contraceptive discontinuation. According to the 2012 DHS, more than half of the women using injectables, male condoms, and oral contraceptives discontinued use of their method within 1 year.^1^ The most frequent reason for discontinuation was the desire to become pregnant (33%), followed by method failure (18%), side effects or health concerns (15%), and the desire to have a more effective method (13%).^1^ Studies have shown that misconceptions about methods persist. A 2015 study highlighted that only two-thirds (65%) of women in Jordan thought that modern methods were more effective than traditional methods, reflecting a lack of accurate knowledge about family planning methods, which may further fuel the use of traditional methods in Jordan.^14^ Given the limited method mix and high rates of discontinuation described above, our assessment suggests that low *contraceptive effectiveness* is the key determinant of fertility stagnation in Jordan.

Low contraceptive effectiveness is likely the key determinant of fertility stagnation in Jordan.

#### Indirect Social Determinants of Fertility

**FIGURE 3.**
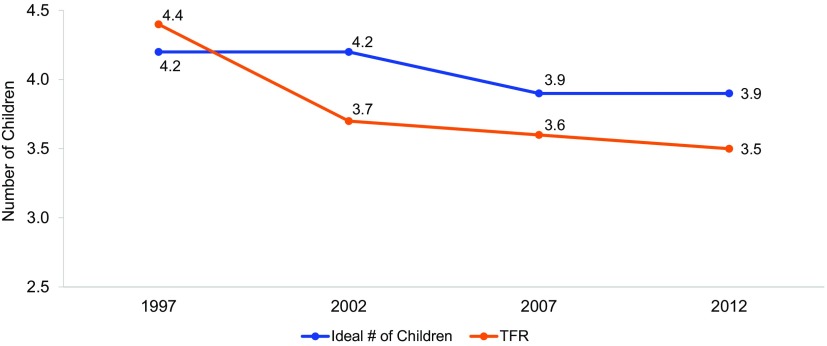
Changes in TFR and Mean Ideal Number of Children in Jordan, 1997–2012 Abbreviation: TFR, total fertility rate. Data from Jordan's Demographic and Health Surveys.

In Jordan, the cultural and social normative factors influencing contraceptive use and effectiveness include expectations around family size and preference for sons, pressure to have a child immediately after marriage, and diffusion of incorrect information about contraceptive methods and their side effects.^15^^–^^19^ Figure 3 highlights the persisting norms around fertility desires: the ideal number of children has declined only slightly, from 4.2 in 1997 to 3.9 in 2012, a decrease that parallels the TFR stagnation. The desire for 4 children is nearly ubiquitous among Jordanians. As a key informant interview participant affirmed: “You want 2 boys, because 2 is better than 1, and 2 girls so they can keep each other company.”

Similar to other contexts, pregnancy and fertility are perceived as a means of upward mobility and status for women in Jordanian society.^16^ Ethnographic research conducted on motherhood in Jordan suggests that reproductive power is generationally passed down from mothers-in-law to daughters-in-law. As young Jordanian women become future mothers-in-law, they may apply the same authority and control they experienced as a newlywed to their daughters-in-law.^16^ Key informant interviewees repeatedly confirmed that both husbands and mothers-in-law can be equally influential in determining use of family planning, including type of method used and continuation or discontinuation.

Pregnancy and fertility can be perceived as a means of upward mobility and status for women in Jordan.

The reproductive power of women in Jordan is inevitably linked with their participation—or lack thereof—in the professional workforce. Despite high education levels, the annual Employment and Unemployment Survey in Jordan showed only a small increase in the participation of women in the labor market, from 12.4% in 1993 to 14.7% in 2011.^7^ The National Reproductive Health/Family Planning Strategy 2013–2017 notes the strong relationship between TFR and women's participation in the labor market, showing that a 1% increase in women's participation in the labor market reduces the TFR by 0.5%.^7^ Other studies in Jordan have come to similar conclusions; for example, a 2012 study asserted that “by limiting women's employment options, the society effectively encourages reproduction, an area where women receive positive social and familial reinforcement,”^20^—a theme that also resonated during key informant interviews.

Overall, the assessment findings showed that the limited method mix, combined with sociocultural determinants of reproduction and fertility desires, have contributed to low contraceptive effectiveness in Jordan.

### What Effect Did USAID Programs Have on Jordan's Family Planning Outcomes?

**FIGURE 4.**
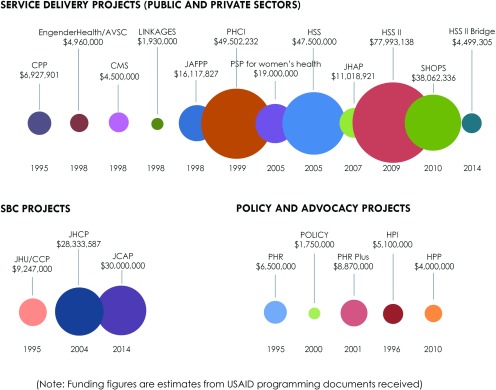
USAID Investment in Family Planning Projects in Jordan, 1995–2015 (in US$ estimates) Abbreviations: AVSC, Association for Voluntary Surgical Contraception; CMS, Commercial Market Strategies; CPP, Comprehensive Postpartum Project; HPI, Health Policy Initiative; HPP, Health Policy Project; HSS, Health Systems Strengthening; JAFPP, Jordanian Association for Family Planning and Protection; JCAP, Jordan Communication, Advocacy, and Policy Activity; JHAP, Jordan Healthcare Accreditation Project; JHCP, Jordan Health Communication Partnership; JHU-CCP, Johns Hopkins Center for Communication Programs; PHCI, Primary Health Care Initiative; PHR, Partners for Health Reform; PHR Plus, Partners for Health Reform Plus; PSP, Private Sector Project for Women's Health; SBC, social and behavior change; SHOPS, Strengthening Health Outcomes through the Private Sector; USAID, United States Agency for International Development. Projects are grouped by category (i.e., service delivery, social and behavior change, and policy and advocacy) and then listed in chronological order based on date of project start; estimated project budgets are in US dollars.

USAID is a key donor to reproductive health programming in Jordan. Exact numbers are not readily available, but a donor landscape report from the Henry J. Kaiser Family Foundation estimated that between 2009 and 2011, USAID provided up to 81% of all foreign government donor funding in reproductive health and family planning.^21^ Figure 4 shows estimated distribution allocations from the 20 USAID projects identified during our desk review from 1995 to 2015, based on project documentation provided by USAID/Jordan. The portfolio distribution shows that USAID reproductive health investments in Jordan have largely focused on service delivery programs, constituting about 75% (US$281.9 million) of USAID investments in reproductive health programming (both public and private). The focus on SBC projects and policy and advocacy projects has been less intensive, with investments totaling 18% (US$67.6 million) and 7% (US$26.2 million) of total USAID investments in reproductive health and family planning in Jordan, respectively.^22^

USAID has largely focused on service delivery programs, constituting about 75% of its investments in reproductive health programming in Jordan.

A comprehensive listing and review of projects according to the 3 investment streams—service delivery, SBC, and policy and advocacy—are provided elsewhere.^8^ For the purposes of this article, however, we highlight key programmatic contributions related to the following categories: (1) increasing access to and use of family planning services, (2) task shifting family planning counseling and services, (3) addressing provider behavior, (4) expanding the method mix, and (5) increasing community demand and support for family planning.

#### Increasing Access to and Use of Family Planning Services

The assessment results showed improvements in the quality of services and access to reproductive health services from facilities at the tertiary, secondary, and primary levels. Improvements include training and capacity development interventions, establishing health facility and community linkages, and renovating health facility structures and investments in medical devices and infrastructure.

In 2011, 439,439 counseling visits for family planning and reproductive health services were conducted in the public sector, increasing to 550,470 counseling visits by 2014.^23^ In particular, the reactivation of community health committees and community awareness activities, under the Health Systems Strengthening II Bridge (HSS II Bridge) project's improvement collaborative, generated immediate demand for family planning services. The exclusive focus on family planning in HSS II Bridge led to reported gains: within 6 months, CYP increased by 50% in the targeted health facilities.^24^

**FIGURE 5.**
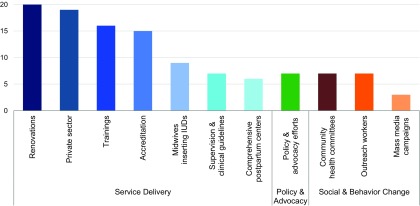
Number of Times Family Planning Successes Were Attributed to USAID Across All Key Informant Interviews (N=69) Abbreviations: IUD, intrauterine device; USAID, United States Agency for International Development.

Key informants commonly attributed increases in access to and use of family planning to USAID-funded programmatic efforts. In particular, key informants cited the contributions of renovation efforts, as shown by the frequency of mentions during interviews that renovations were successful (Figure 5). For example, key informants said that renovations of hospitals' comprehensive postpartum centers in the late 1990s increased access to postpartum family planning services by providing separate, private family planning counseling rooms.

#### Task Shifting of IUD Insertion Services

Jordan has a shortage of female medical providers given women's limited participation in the workforce. In response, Jordan's Higher Population Council, with support from USAID, advocated for shifting the task of IUD insertions and removals to midwives within MOH facilities. However, this effort was constrained by national policy legislation. In 2010, Jordan's MOH temporarily banned midwives from inserting IUDs.^25^ The ban was eventually overturned due to efforts from the Higher Population Council and USAID. After midwives began inserting IUDs again, MOH CYP increased slightly from 119,995 in 2011 to 126,696 in 2014.^25^ Concurrently, the number of MOH health centers providing IUDs each year increased from 88 to 160 health centers between 2011 and 2014.^18^ Staff turnover was also reported as a barrier to providing family planning services. During interviews, health service providers in primary health centers and hospitals expressed frustration at the departure of midwives previously trained by USAID in IUD insertion and family planning counseling.

Evidence also suggests that decentralizing family planning counseling to community outreach workers can be effective in lowering the use of traditional family planning methods. A 2015 study in Jordan evaluating a home visit outreach voucher program conducted by social workers showed 48% and 59% gains in modern method uptake among women-only counseling and couples-counseling intervention groups, respectively, relative to control. The study, evaluated under the Strengthening Health Outcomes through the Private Sector (SHOPS) project, also showed lower use of traditional methods and fewer concerns about side effects in both women-only counseling and couples-counseling intervention groups.^26^

#### Addressing Provider Behavior

The assessment findings suggest that both public- and private-sector providers have misconceptions about hormonal methods and side effects, despite repeated USAID investment in training initiatives.^27^^–^^29^ One initiative included the evidence-based medicine program, a 3-month education and training program for providers on injection counseling, conducted by the SHOPS project. A 2014 randomized study showed that evidence-based medicine training provided to private-sector physicians improved their attitudes and confidence in providing injectables, but did not improve knowledge of the method or counseling practice scores.^27^

In contrast, the “Consult and Choose” initiative, under the Jordan Health Communication Partnership, pilot-tested a client-centered family planning program in the governate of Irbid, synchronizing service delivery and community outreach activities to increase access to family planning services among women with a high unmet need. The study results showed that 83% of clients reported being “very satisfied” with their counseling visit, with health care providers completing 5.6 of the 7 counseling protocol steps. Descriptive results also suggested an increase in new family planning users and CYP after program start.^30^

#### Expanding the Method Mix

A few projects, such as SHOPS, have attempted to expand the method mix and access to underutilized methods in Jordan, including injectables and implants. Although a number of methods have been introduced in Jordan, methods such as injectables and implants are still not routinely or widely available. Under the HSS II project, the number of MOH primary comprehensive health centers offering 4 or more family planning methods increased from 106 to 145 health centers between 2011 and 2014.^18^ At the time of the assessment, however, few health centers offered more than 4 methods, and some key informants reported frequent stock-outs of implants and injectables.

#### Increasing Community Demand and Support for Family Planning

Social communication strategies in the late 1990s were instrumental in garnering social support for family planning, including mobilizing religious leaders to support family planning as a method of child spacing. Mass media campaigns targeted husbands and mothers-in-law, and other familial networks who played an influential role in disseminating information about family planning methods and who pressured for pregnancies soon after marriage. For instance, the “Together for a Happy Family” campaign targeted more than 2 million people and was the first-ever national campaign to specifically target men. During the campaign from 1996 to 2000, the number of surveyed men who considered IUDs safe for their wives rose from 34% to 50%, and the number of men who considered the pill safe increased from 25% to 36%.^31^

More recent mass media campaigns have shown mixed results in sustaining family planning demand. Evaluation results of the SHOPS social marketing campaign from 2011 to 2013 showed that 91% of respondents remembered the campaign slogan when prompted. The evaluation results also showed that the likelihood of being an oral contraceptive user increased with increased exposure to the campaign. While evidence suggests that the SHOPS social marketing campaign for IUDs boosted the uptake of IUD services, demand dropped after the campaign ended.^32^

#### Enabling a Supportive Policy Environment for Family Planning

Two key components of USAID/Jordan's strategy for family planning include supporting (1) Jordan's policy environment and implementation of the reproductive health/family planning strategy and (2) the capacity of local partners, such as the Higher Population Council, to advocate and conduct awareness-raising and policy reform activities.^33^ Key contributions include supporting the development of a contraceptive security strategy to support the national population strategy, creating several national and MOH reproductive health and family planning strategies (e.g., MOH Strategy 2008–2012, MOH Strategy 2013–2017, and MOH Family Planning Strategy 2013–2017), and increasing the legal age of marriage from 15 to 18 for women and 16 to 18 for men, through the POLICY project among others.^7^

Given Jordan's competing priorities, and the growing influx of refugees, financial commitment from the central government toward family planning programs is waning. The government's limited financial commitment and ownership affects the potential sustainability and scale-up of effective family planning programs. At the time of the assessment, key informants commonly reported that scale-up efforts were limited, and effective pilot projects were often discontinued despite reported gains. These include successful initiatives such as the “Consult and Choose” client-centered counseling pilot study and family planning awareness-raising activities conducted by community health committees under the service delivery projects to generate local family planning demand.

The government's limited financial commitment and ownership affects the potential sustainability and scale-up of effective family planning programs in Jordan.

## DISCUSSION: MOVING FORWARD WITH FAMILY PLANNING PROGRAMMING IN JORDAN

The examination and analysis of 2 decades of family planning programs in Jordan provides an opportunity to make recommendations for future programming. Stakeholders in Jordan have expressed interest in increasing the scale and sustainability of programs and capitalizing on recent reproductive health gains. Fertility stalls are not uncommon and some lessons can be extracted from other countries. For example, in 2005 USAID studied family planning stalls in Senegal^34^ and Tanzania,^35^ both of which have since seen CPR increases. In both countries, increased government commitment to family planning was a key factor in improving outcomes. In particular, Senegal followed through on its 2012 London Summit on Family Planning commitment to double their domestic investment in health.

Expanding the method mix should be a government priority in Jordan. In 2010, the Higher Population Council, with support from USAID's Health Policy Initiative, conducted a simulation to assess how changing the method mix would affect TFR. The results showed that reducing traditional method use (e.g., withdrawal) by half could contribute to reducing TFR by 0.35 points, from 3.80 to 3.45 children per woman. Desk review findings highlight 3 issues that need to be prioritized to improve the method mix: (1) fear of side effects/health concerns, (2) provider behavior and bias, and (3) female provider availability.^36^

Programs addressing cultural norms around family planning and fertility desires also need to be prioritized. A 2011 USAID-funded SBC program report concluded that access, cost, and quality of care are not the main constraints to adoption and use of contraception in Jordan. The authors stated that “improved quality of care can affect fertility timing, but will not change fertility levels without a change in desired fertility.”^37^ Beyond the mass media campaigns described in this article, there have been less investment and programming in SBC activities that can address the strong social norms among close-knit families and communities concerning large family size and son preference.

SBC activities should, and can be, synchronized with service delivery programming. As some evidence supports, strengthening effective and sustainable health systems cannot occur without systematically integrating behavior change approaches within these health systems.^38^ A few notable models exist, including the Health Communication Capacity Collaborative's Circle of Care Model, a programmatic framework that interweaves SBC programming along the service delivery continuum.^39^ Specifically, this service delivery continuum offers 3 points of contact—before, during, and after services—to influence attitudes and behaviors of both clients and providers.

The Syrian refugee crisis was a recurring, looming topic during key informant interviews that should be addressed. The exact impact of the Syrian refugee influx on national CPR and TFR outcomes is unknown. Some key informants in Jordan shared perceptions that the new Syrian refugees bear more children, do not believe in family planning methods, and are polygamous. Yet, findings from a 2016 qualitative gender study conducted by the Jordan Communication, Advocacy, and Policy (JCAP) project showed that Syrian and Jordanian women have similar preferences for family size and desired number of children.^16^ Some key informants cited the influx of Syrian refugees as a reason for TFR stagnation. However, this is more a reflection of perceptions and tensions around the refugee situation than actual causality, as the stagnation predated the latest influx of refugees.^40^

It is possible that some improvements in TFR and mCPR occurred from 2012 to 2015, which are not captured in the latest DHS data. In fact, a number of interviewed Jordanian stakeholders rebuked the existence of a TFR stall, pointing to several improvements in family planning outcomes over the last 2 decades. Interestingly, a new calculation indicates that there might have been a TFR reduction in the last few years, estimated as 3.1 for 2014.^41^ This new calculation, based on Schoumaker's model,^42^ uses data from birth registries, which is considered a strong data source because 99% of women in Jordan give birth in health facilities. One strength of this measure is that it can be calculated more regularly, rather than waiting 5 years for each DHS study. USAID and the MOH have accepted the new TFR measure as accurate, but at the time of the field interviews in early 2016, it was unclear how accepted it would become within the wider body of stakeholders in Jordan. One challenge with this new calculation is that it includes only women of Jordanian nationality or who are registered as living in Jordan, which leaves out a large and growing refugee population.

Some improvements in total fertility and use of modern contraceptives may have occurred from 2012 to 2015; however, the new calculation does not include a portion of the refugee population.

Our additional analysis of CYP data collected from the online MOH logistics system, which covers the public sector and most of the NGOs receiving MOH contraceptives, confirmed a similar stalling in line with the DHS data, from 227,531 CYP in 2012 to 227,600 in 2015.^25^ One issue with this measure is the quality and reliability of the data, which is shared through an online MOH logistics system, after collection from local health facilities. Finally, our review of USAID programs included at least 2 projects (HSS II Bridge and JCAP) that began after the last recorded DHS period, and therefore the effect of these programs on family planning outcomes cannot be fully ascertained.

## CONCLUSION

The government of Jordan, with support from USAID, has made substantial progress in increasing access to and use of family planning services. However, much more can be done to address the limited method mix and sociocultural determinants of contraceptive use. In contexts where sociocultural norms are the dominant barriers to use of family planning, increased investments in SBC and advocacy programming may be needed to catalyze behavior change toward improved and sustained family planning use.

Lastly, while we use the Jordan case study as an example of a family planning programming approach in the context of a fertility stall, we recognize that shifting gender and social norms around fertility intentions can be difficult, intensive, and contentious. Addressing such influential norms requires the help of practitioners, researchers, and donors to critically unpack what we can and cannot change about values and belief systems deeply rooted within social norms and expectations around pregnancy and family aspirations. Ultimately, programmatic activities that address community and familial fertility norms, increase family planning options available to couples, and empower governments to implement and sustain project activities will contribute to an evolving landscape of improved reproductive health service delivery, and will enable countries like Jordan to meet their fertility goals.

## Notes

**Peer Reviewed**

**Funding:** USAID.
